# Thermodynamic and Kinetic Study of Diels–Alder Reaction between Furfuryl Alcohol and *N*-Hydroxymaleimides—An Assessment for Materials Application

**DOI:** 10.3390/molecules25020243

**Published:** 2020-01-07

**Authors:** Jamerson Carneiro de Oliveira, Marie-Pierre Laborie, Vincent Roucoules

**Affiliations:** 1 Chair of Forest Biomaterials, Faculty of Environment and Natural Resources, University of Freiburg, 79085 Freiburg, Germany; jamerson.oliveira@biomat.uni-freiburg.de (J.C.d.O.); marie-pierre.laborie@biomat.uni-freiburg.de (M.-P.L.); 2 Freiburg Materials Research Center, University of Freiburg, 79104 Freiburg, Germany; 3 IS2M, UMR 7361, CNRS, Université de Haute-Alsace, Université de Strasbourg, F-68100 Mulhouse, France

**Keywords:** Diels–Alder, materials, kinetic, thermodynamic

## Abstract

The study of Diels–Alder reactions in materials science is of increasing interest. The main reason for that is the potential thermoreversibility of the reaction. Aiming to predict the behavior of a material modified with maleimido and furyl moieties, ^1^H NMR and UV-Vis solution studies of the Diels–Alder reaction between furfuryl alcohol and two *N*-hydroxymaleimides are explored in the present study. Rate constants, activation energy, entropy, and enthalpy of formation were determined from each technique for both reacting systems. *Endo* and *exo* isomers were distinguished in ^1^H NMR, and the transition from a kinetic, controlled Diels–Alder reaction to a thermodynamic one could be observed in the temperature range studied. A discussion on the effect of that on the application in a material was performed. The approach selected considers a simplified equilibrium of the Diels–Alder reaction as the kinetic model, allowing materials scientists to evaluate the suitability of using the reacting molecules for the creation of thermoresponsive materials. The proposed approach determines the kinetic constants without the direct influence of the equilibrium constant value, thereby allowing a more objective data analysis. The effects of the selection of kinetic model, analytical method, and data treatment are discussed.

## 1. Introduction

Diels–Alder (DA) reaction is a well-known [4+2]cycloaddition between a conjugated diene and a dienophile. That addition reaction generally produces two diasteroisomers (*endo* and *exo*). The formation and the proportion of the isomers depend on several conditions such as the solvent, temperature, and catalyst [1,2]. The study of Diels–Alder reaction kinetics is extensively explored in literature [3,4,5]. Depending on the diene and dienophile selected and the reaction conditions, Diels–Alder reaction can be considered irreversible—meaning that the retro-Diels–Alder reaction is neglectable. That consideration is used in several publications [6,7,8,9]. The complexity of the kinetic model is increased when the reaction equilibrium and isomers are taken into account [10]. The possible application of the Diels–Alder/retro-Diels–Alder in responsive materials resulted in greater interest for the consideration of the equilibrium reaction in kinetic models for materials applications [11,12,13,14,15].

The use of Diels–Alder reaction in materials science is not a new research topic [16], and nowadays, it covers a significant range including polymer synthesis, pharmaceutical, and biomedical engineering [17,18,19,20,21]. In that field, the complication of considering the equilibrium reaction with the kinetic model of the Diels–Alder reaction can be often minimized, depending on the system studied and the methodology selected. For example, Vauthier et al. [9] evaluated the reaction between a plasma polymer surface functionalized with furan moieties and maleic anhydride in solution. In their study, the authors found that the consideration of an equilibrium reaction did not considerably change the values obtained for the rate constant of the forward Diels–Alder reaction. That could be explained by the fact that the amount of species present in the solution was much higher than in the surface. However, in some cases, it might be necessary to consider the influence of the retro-Diels–Alder on the kinetics of the system. That is true for the case of materials that will react in near-equimolar proportions or in which the reversibility is planned to take place in near-equilibrium conditions. Diaz et al. [14], Stirn et al. [12], and Koehler et al. [11] modeled the kinetics of their system through different approaches, but considered an equilibrium reaction between diene, dienophile, and one adduct.

Another factor to be considered in the case of evaluation of the Diels–Alder reaction and the reversibility for materials application is the ratio between the isomeric products. As the *endo* and *exo* products are known to undergo the retro-Diels–Alder reaction at different temperatures, the application of that reaction in a responsive material—that ideally will operate in cycles—should be carefully analyzed. Some works have been dedicated to that issue. Froidevaux et al. [13] chose a kinetic approach that minimized the effect of the equilibrium reaction by selectively analyzing the *endo* adduct decomposition reaction. In a solvent-free approach, Cuvellier et al. [15] showed a strong agreement between a kinetic model used to determine *endo*/*exo* composition, obtained via calorimetric experiments, and experimental results obtained via ^1^H NMR.

The present work was oriented by the idea of predicting the Diels–Alder/retro-Diels–Alder reaction conditions for use in a suspended material. The fact that the suspension should be analyzed in near-equilibrium conditions requires the prediction of not only the kinetic, but also the thermodynamic behavior of the reaction. Although the thermodynamic behavior of the suspended particles could be the limiting factor for the prediction of the suspension behavior, the present study provides information necessary to prove or discard that hypothesis. The approach selected in the present work aims to debate qualitatively the influence of *endo*/*exo* isomers on the reversibility of the reaction. It also proposes a method for the determination of the kinetic and thermodynamic parameters that, although the *endo* and *exo* products are not considered in the model, offers the possibility of a more precise determination of those values than similar contributions in the field.

## 2. Results and Discussion

**Scheme 1:**
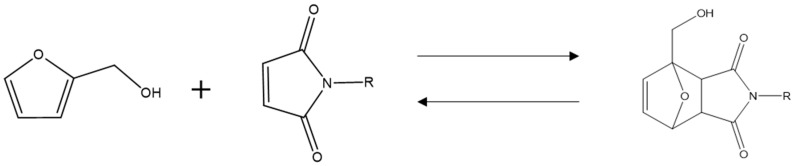
Reaction of furfuryl alcohol (FA) and selected maleimides. R = OH. R = CH_2_CH_2_OH.

The current work evaluated the kinetic and thermodynamic parameters of two different Diels–Alder reactions. Hydroxyl terminated molecules were selected due to the possibility of using that function for future grafting onto the particles of the suspension. The reactions studied here were between furfuryl alcohol (FAL) and two *N*-hydroxymaleimides: *N*-Hydroxymaleimide (NHM) and *N*-(2-Hydroxyethyl)maleimide (N2HM). The following Scheme 1 shows the chemicals and the expected reaction product. Along with the present work, we explored the importance of understanding the reactivity and equilibrium conditions of the molecules. The reaction between FAL and NHM is further referred to as FAL–NHM reaction, whereas the reaction between FAL and N2HM is referred to as FAL–N2HM.

### 2.1. Importance of the Analytical Method and Influence of the Product for Materials Application

Maleimide disappearance over time could be followed by means of UV-Vis spectroscopy. From this data, estimation of product formation was done by considering the absence of any side reaction. Another important limitation of the methodology used in the UV-Vis interpretation is that we considered that the maleimide was the only component absorbing at a wavelength of 340 nm, selected for the data treatment. A similar approach was reported by Gandini et al. [22] when evaluating the polycondensation of furan–maleimide monomers. The authors attribute the observed decrease in absorbance to the loss of conjugation between the carbonyl groups and the unsaturation present in the maleimides.

It might interest the reader to know that several trials of curve calibration considering all the four reactants and products (diene, dienophile, *exo*, and *endo* products) were performed. However, due to difficulties regarding purification—especially in the isolation of the *exo* and *endo* products—the data obtained with these calibrations were not better than those obtained through the method described before.

**Figure 1:**
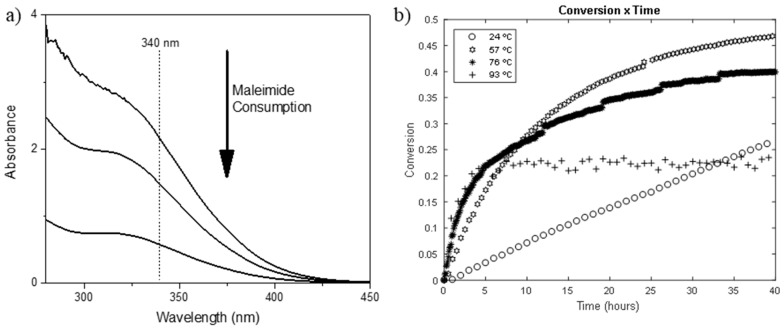
(**a**) UV-Vis spectra showing the decrease of absorbance attributed to the disappearance of *N*-hydroxymaleimide (NHM) when reacted with furfuryl alcohol at room temperature, and (**b**) plot of product conversion vs. time for different reaction temperatures for the same reacting system obtained via UV-Vis.

Figure 1a shows representative spectra collection for the UV-Vis experiment at different times of the reaction. Figure 1b clearly shows the effect of temperature on the kinetic and thermodynamic of the reaction. For the reaction at 24 °C, a linear behavior is observed within 40 h, indicating that the system is far from equilibrium, while for the reaction at 93 °C, a plateau was reached before 5 h of reaction, indicating that equilibrium was already reached. It is also possible to verify the influence of the temperature on the final conversion of the reaction, with the reactions at lower temperatures resulting in higher conversions, as expected. The same data analysis from the FAL–N2HM reaction shows a similar trend to the one already discussed so far (Figure S1). The main difference observed lies in the time for achieving equilibrium, which was found to be longer in the case of the latter.

When analyzing the effect of maleimide substituents on the reaction reversibility and kinetics, Boutelle and Northrop [23] concluded that, although the reaction kinetic does not significantly change with the dienophile substituent, when compared to the impact of substitution in the diene pair, a slight increase in the thermodynamic favorability of the adduct formation occurs. The importance of that analysis for a material behavior prediction comes from the fact that generally the molecules studied for the prediction contain functionalities (or chain length) other than after their reaction with the surface. Furthermore, the proximity of those molecules to the surface could also influence reactivity in accordance with the chemistry and mobility of the surface. Those factors should be considered for the prediction of the reaction in the interface. Otherwise, the expected reaction time or conversion could be very different to that of the solution.

**Figure 2:**
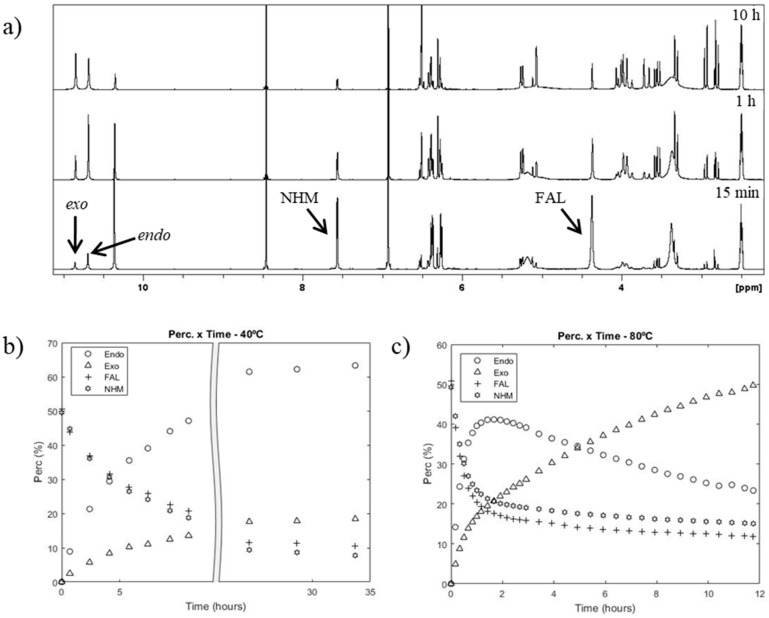
(**a**) Proton NMR spectra of the furfuryl alcohol/*N*-Hydroxymaleimide (FAL–NHM) reaction at different times and at 60 °C; (**b**) Percentage (%) versus time (hours) of FAL–NHM reaction at 40 °C; (**c**) Conversion (%) versus time (hours) of FAL–NHM reaction at 80 °C.

^1^H NMR allowed us to follow not only the conversion time of both reactants into the adduct, but also the separation of the latter into *exo* and *endo* isomers (Figure 2). Other advantages of the technique lie in the identification of possible side reactions and the possibility to access the quality of sample preparation via differentiation of the reactants.

From the ^1^H NMR analysis, it was possible to confirm that no side reaction was present in all systems. In Figure 2a, the three most downfield peaks (higher than 10 ppm) correspond to the hydroxyl peaks of maleimide (10.34 ppm), *endo* (10.66 ppm), and *exo* (10.82 ppm) adducts. Although not used for quantification, they clearly illustrate the reaction progress over time. As observed, ^1^H NMR allowed the evaluation of the variation of *endo* and *exo* products concentration. One can infer from the data set (Figure S2) that thermodynamic reaction control should be considered for FAL–NHM for temperatures at 60 °C or above, while for temperatures at 40 °C or below, kinetic control is dominant. For lower temperatures, there is a clear preference towards the formation of the kinetic product (*endo*) with no notable conversion of this product to the thermodynamic one (*exo*). For higher temperatures, at 60 °C or above, it is possible to notice that, after some time, there is no significant conversion of reactants into products, but that a transformation of the *endo* into the *exo* isomer still occurs. This is well illustrated in Figure 2c where, after achieving a “peak concentration” after two hours of reaction, the amount of *endo* product starts to decline, while the amount of *exo* isomer increases. Rulísek et al. [10] observed that the barrier for direct conversion of *endo* to *exo* adduct is too high for the reaction to happen. The conversion of one product to the other thus occurs through a first reverse reaction followed by a forward one. The authors explain that the low thermodynamic stability of furan favors the occurrence of retro-Diels–Alder and thus the conversion of the kinetically favored product to the thermodynamically favored one.

Froidevaux et al. [13] performed a mindful work on analyzing Diels–Alder reaction for materials application. The authors’ kinetic approach minimized the effect of the equilibrium reaction by selectively analyzing the *endo* adduct decomposition reaction. Although the approach of the authors constitutes a careful study, it limits the prediction of the behavior of the material—once only the decomposition of the *endo* isomer is taken into account. For the idea of a Diels–Alder reaction happening between particles in suspension, considering that the mobility of suspended particles allows the variation in *endo*/*exo* ratio according to the applied temperature, the optimization of the temperature for the retro-Diels–Alder could not be based only on the selection of the *endo* product decomposition. The increase in temperature actually would have led to the conversion of the *endo* to the *exo* isomer—with the transition of the kinetic control of the reaction to the thermodynamic one. A practical effect of this change for an application in a material is that, although the increase in temperature would have led to a decrease in the overall equilibrium of the Diels–Alder reaction, the conversion of the *endo* to the *exo* isomer would have increased the temperature, to which further change of the system could have been observed. There would also have been an issue if the material was designed to undergo several cycles. In that case, a material that was produced with the intent of optimizing the *endo* adduct ratio would have had a lower ratio of that adduct after heating and returning to room conditions.

A model considering several reactions in parallel would have best represented the reacting system that we proposed to study. However, it would have complicated the prediction and required a greater amount of experiments. Due to that, the use of a simplified equilibrium reaction was selected. As is discussed later, the equilibrium selected does not completely eliminate all the issues, but it offers a reliable and replicable approach. In terms of estimation of the material behavior, the information obtained by the techniques selected are highly valuable. The use of two techniques that considerably differed in the initial concentration highlighted that that concentration should also be considered for the application of the Diels–Alder in a material. For example, in the UV-Vis experiment, the equilibrium conversion of the FAL–NHM adduct was about 40% at 76 °C, while in the ^1^H NMR experiment, the conversion was over 70% at 80 °C. Thinking again about a suspension of reacting particles, with the hypothesis that the reaction kinetic is the limiting factor for the conversion, ^1^H NMR and UV-Vis become complementary techniques, as they offer the estimation of the conversion in a broad concentration range. All the above information is useful for establishing the initial conditions for the material analysis. The kinetic and thermodynamic parameters of the reaction are summarized below. A focus on the importance of the methodology selected for that is discussed, and a suggested approach for the data treatment is presented.

### 2.2. Data Fitting and Determination of Rate and Equilibrium Constants

The kinetic data treatment was based on what is called a complicated rate equation, more specifically the case of a reversible reaction:

$$A+B\begin{array}{c} \overset{k_{f}}{\to }\\ \underset{k_{r}}{\leftarrow } \end{array}C$$

Assuming that the reaction is elementary, we can write the rate equation:

$$\frac{dA}{dt}=-k_{f}\left[A\right]\left[B\right]+k_{r}\left[C\right]$$

The premise that the Diels–Alder reaction is elementary is common in publications and, of course, agrees with its commonly assumed concerted mechanism. Another important assumption, for writing the rate equation as shown above, is that there is no distinction between the products of the Diels–Alder reaction (*endo*/*exo* isomers). Thus, they are considered a single product of the reaction. The last supposition could be problematic, but the consideration of two (or more) reversible reactions happening in parallel falls outside the idea of having a simpler way of vefying the possible applicability of a reaction for a material. Furthermore, recent work from Cuvellier et al. [15] showed that, for a model considering the *endo* and *exo* adduct, only slight differences in the calculated kinetic and thermodynamic values were observed when compared to a model considering only one product. The authors, however, observed that the use of two parallel equilibrium reactions modelled the reaction better at higher temperatures. An integrated solution of the above rate equation was provided by Benson [24] as:

x=(β+q122γ)etq12+θ−(β−q122γ)1−etq12+θ

where: *t* is time, x=[A]0−[A], θ=lnβ−q12β+q12, $q=\beta^{2}-4\alpha \gamma$, $\alpha =k_{f}{\left[A\right]}_{0}{\left[B\right]}_{0}-k_{r}{\left[C\right]}_{0}$, $\beta =-\left[k_{f}{\left[A\right]}_{0}+k_{f}{\left[B\right]}_{0}+k_{r}\right]$, $\gamma =k_{f}$.

Fitting of this solution (conversion as a function of time) was performed with a nonlinear fitting function (lsqcurvefit) in Matlab. After fitting, the kinetic and thermodynamic parameters of the reaction FAL–NHM were obtained from both techniques used (Table 1).

As expected, with the increase of temperature, both kinetic constants increase and the equilibrium constant decreases. The Sum of Squared Residuals (SSR) shows that all the fitting performed agrees well with the data obtained, with slight variations among measurements from the same technique. A closer look at the fitting and the data points at different temperatures (Figures S3 and S4) shows that for higher temperatures, especially at 100 °C, there is a less precise prediction of the kinetics when equilibrium is nearly reached. This is caused by the limitation of the selected kinetics, which does not separate the two adducts into two different equilibrium reactions. Increase in prevalence of the thermodynamic control results in a less trustworthy prediction, as the hypothesis of only one reaction happening becomes less realistic. That result agrees well with what was reported by Cuvellier et al. [15].

To the best of our knowledge, there are no reported data of the two reactions explored here, preventing the comparison of absolute values of rate constants to literature. It is also important to have in mind that the solvent (deuterated dimethyl sulfoxide (DMSO-*d*_6_)) might significantly influence the reaction rate calculated here, because hydrogen bonding is known to have an effect on Diels–Alder reaction rates [25].

When comparing rate constants to literature, obviously, consideration should also be given to the kinetic approach selected by each report. Most of the literature consulted uses a kinetic approach that neglects the influence of retro-Diels–Alder reaction [26,27,28]. However, when the reversibility of the reaction on materials application is an issue of the publication, the kinetic study becomes generally more complex and closer to the one used here. For instance, Stirn et al. [12] and Koehler et al. [11] used the same approach as the present work in terms of differential rate law. The difference to these works lies in the method selected for the data treatment. Both publications selected the calculation of the equilibrium constant prior to the determination of the kinetic parameters. Although the final equations and method for determination of the kinetic rate constants varied between them, both publications needed the value of the equilibrium constant to calculate their kinetic rate constant. In the present data treatment, the approach selected determines the kinetic constants without the direct influence of the equilibrium constant value—which is calculated here as the ratio between forward and reverse rate constants. The issue in the method reported previously in literature is that the selection of one point (or even several) to determine the value of the equilibrium constant carries more error than if all the data points collected are used instead. Because the FAL–NHM and FAL–N2HM reactions needed a long time to achieve equilibrium, especially at lower temperatures, it was hard to choose which points would represent this condition. In other words, the question that the researcher has to decide for each different temperature is: What change in conversion is small enough and in which time scale? “Outsourcing” the decision of the equilibrium to the fitting algorithm does not completely eliminate this question, but at least provides the advantage of making the replication of calculated results more accurate.

For reaction FAL–N2HM, fewer temperatures were studied. The ranges of temperature and time were preselected from the reaction FAL–NHM, and the focus was on reactions happening above 60 °C, due to their shorter time. ^1^H NMR results presented similar trends to what was already discussed, while the values of the equilibrium constant did not always decrease with the increase in temperature (Table 2). The only noticeable difference in the fitting was that the time of the measurement was too short for the measurements at 60 and 80 °C. These results might thus lead to an overestimation of the real value of the rate constant.

The stability of FAL, NHM, and N2HM was previously tested with ^1^H NMR by heating the sample at 130 °C in DMSO-*d*_6_ overnight. It was not possible to see any considered side reaction for all compounds. The results from Table 2 raise the question of whether the conditions of the UV-Vis measurement could have a different effect on the possibility of side reactions. Indeed, when N2HM was heated for several hours at 76 °C, it was possible to see a decrease in the absorbance at 340 nm. This result indicates that a side reaction is happening with N2HM in the conditions of the UV-Vis experiment. One hypothesis is that, even though absolute DMSO was used for the measurement, the amount of water present or incorporated during the measurement was higher than that present in the ^1^H NMR, favoring the occurrence of hydrolysis over the Diels–Alder reaction. In addition, the different concentrations used in each technique completely changed the ratio between the maleimide and water, influencing the kinetics of a probable hydrolysis. Another possible hypothesis is that maleimide photolysis is accelerated by consecutive spectra acquisitions in the UV-Vis.

### 2.3. Calculation of Activation Energy, Entropy, and Enthalpy of Formation

The Arrhenius equation allows the determination of the activation energy of the forward and reverse Diels–Alder reactions as:

$$k=Ae^{\frac{E_{a}}{RT}}$$

where *k* is the rate constant, *A* is the pre-exponential factor, *E_a_* is the activation energy, *R* is the universal gas constant (8.314 J/K·mol), *T* is the reaction temperature.

Linearization of the Arrhenius equation results in

$$\ln k=\ln A-\frac{E_{a}}{RT}$$

**Figure 3:**
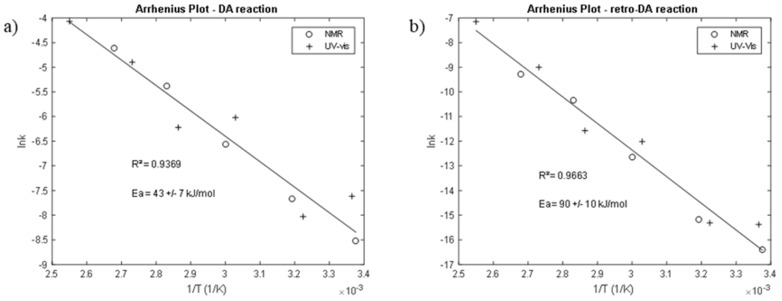
Arrhenius plot for FAL–NHM reaction: (**a**) Forward reaction and (**b**) reverse reaction.

This equation can now be used in an Arrhenius Plot (ln*k* versus 1/*T*), where the value of the slope of the fitted line is equal to the activation energy. Figure 3a shows the fitting, the value of the activation energy, and the coefficient of determination for the forward FAL–NHM reaction. Figure 3b shows the same features for the retro-Diels–Alder reaction. In the graphs, calculated values from both techniques (UV-Vis and ^1^H NMR) are plotted together.

The activation energy value for the forward reaction (43 ± 7 kJ/mol) is lower than that of the reverse reaction (90 ± 10 kJ/mol), as expected. The calculated values of activation energy are in the range of what is generally reported for Diels–Alder and retro-Diels–Alder reactions [11,12]. The results for the reaction FAL–N2HM (Figures S5 and S6) show only the data treatment from the NMR spectroscopy, due to the side reaction observed in the UV-Vis experiment. Activation energy values for the FAL–N2HM reaction were a bit lower than those of FAL–NHM (32 kJ/mol for the forward reaction and 59 kJ/mol for the reverse). This does not agree with the observed slower kinetics for FAL–N2HM. This difference might be the result of the overestimation of the rate constants for the lower temperatures, reported previously. As fewer temperatures were used for this experiment, the standard errors of the estimates were also higher.

Enthalpy and entropy of formation were calculated via the linear form of the van ’t Hoff equation:

$$\ln K=-\frac{\Delta H}{RT}+\frac{\Delta S}{R}$$

**Figure 4:**
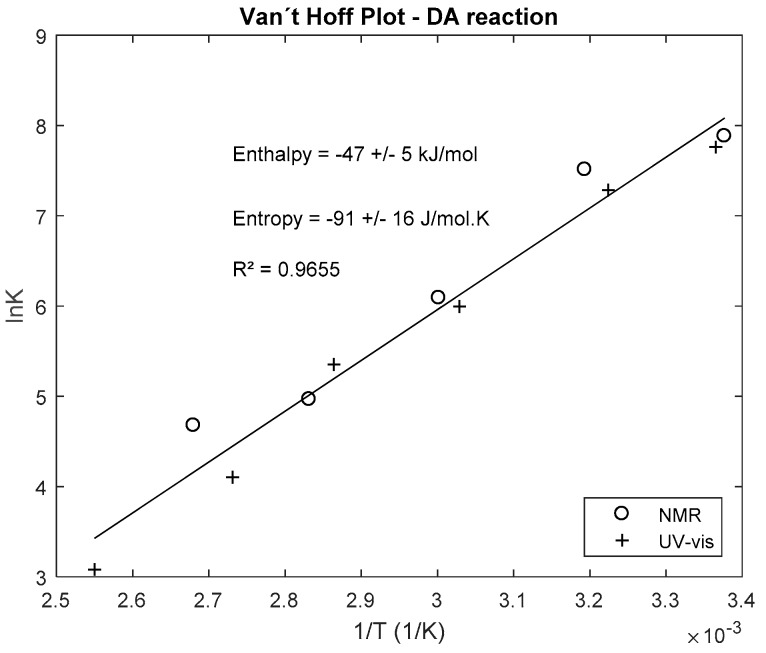
Van ’t Hoff plot for reaction FAL–NHM.

After plotting the logarithm of the calculated equilibrium constants versus the inverse of temperature, the enthalpy of formation correlated with the slope of a fitted straight line, and the entropy with the linear coefficient. The plot for the reaction FAL–NHM is shown in Figure 4.

The values obtained for the thermodynamic parameters are in the same range as for other Diels–Alder reactions. Due to the smaller amount of data collected for the reaction FAL–N2HM, the error in the values obtained for enthalpy and entropy were too big to be statically significant. All the results of the calculated thermodynamic and kinetic data are summarized in Table 3.

It is possible to notice in Table 3 the difference in values obtained via each technique for reaction FAL–NHM. However, it is worth noting, for example, that the effect of temperature in the pre-exponential factor is not considered in the Arrhenius equation, which might have led to the observed difference of the calculated values of activation energy. If the effect of temperature on the pre-exponential factor is taken into account (equation below—for an elementary bimolecular reaction), the activation energy for the NMR experiment becomes 31.51 kJ/mol, while for the UV-Vis, it is 35.36 kJ/mol, reducing the previous difference between the experiments from 8.51 to 3.85 kJ/mol.

$$k=c\times \sqrt{T}\times \exp\left(\frac{E_{a}}{RT}\right)$$

As the linearized form of the van’t Hoff equation also assumes that there is no influence of the change in temperature on the enthalpy, a similar source of error should also be considered when comparing the obtained values.

## 3. Materials and Methods

### 3.1. Materials

Furfuryl alcohol (FAL, analytical standard, Sigma-Aldrich, St. Louis, MO, USA), *N*-Hydroxymaleimide (NHM, 97%, Sigma-Aldrich), *N*-(2-Hydroxyethyl)maleimide (N2HM, 97%, Sigma-Aldrich), 1,2,4,5-Tetrachloro-3-nitrobenzene (NMR Standard, Sigma-Aldrich), and deuterated dimethyl sulfoxide (Sigma-Aldrich) were used as received.

### 3.2. ^1^H Nuclear Magnetic Resonance—^1^H NMR

^1^H NMR was acquired from a Bruker Avance III HD 400 MHz or Bruker DPX 200 MHz NMR spectrometer (Bruker, Billerica, MA, USA). All reactants were premixed in an Eppendorf tube and immediately transferred to an NMR tube, right before acquisition started. This process took around 2 min and was performed to guarantee good mixing of reactants. Deuterated dimethyl sulfoxide (DMSO-*d*_6_) was used as solvent, due to its high boiling. Initial concentration of reactants was 0.172 mmol/mL and 1,2,4,5-Tetrachloro-3-nitrobenzene was used as the internal standard for quantitative analysis. The reactants were mixed in the absence of adducts.

Chemical shifts of all the reactants and products are reported in the Supplementary Information.

For quantitative purposes, the spin–lattice relaxation times (T1) of the samples were measured through a standard inversion recovery experiment. All T1 values observed were under three seconds. A recycle delay (D1) of 15 s was used in the experiments.

### 3.3. UV-Vis Spectroscopy

UV-Vis spectra were acquired in a Lambda 35 UV/VIS spectrometer from PerkinElmer (Beaconsfield, United Kingdom). Temperature control was done with a FalconTM Heated Transmission Accessory from Pike Technologies (Madison, WI, USA). Acquisition was done in the range of 450 nm to 265 nm with a resolution of 1 nm. Diene and dienophile were mixed in a 1 cm path length quartz semi-micro cuvette with a stopper. Acquisition started right after mixing. Absolute DMSO was used as solvent, and the initial concentrations of the diene and dienophile were always 5.3 × 10^−3^ mmol/mL. The reactants were mixed in the absence of adducts.

## 4. Conclusions

The influence of the selected technique and especially of more subjective decisions, such as duration of experiment and methods of calculation, were discussed. We advise the use, whenever possible, of ^1^H NMR following the reaction. The advantage of the technique relies on the possibility of identification of the isomers and of side reactions. Other techniques, such as UV-Vis, could be used as a complementary approach. Although the selected kinetic model does not include parallel equilibrium reactions, the methodology used for the calculation of the parameters is more reliable and less subjective than those commonly used by materials scientists when modeling Diels–Alder reactions in solution. For the reaction FAL–NHM, it was possible to observe a decrease of 20 times in the equilibrium constant when comparing experiments at room temperature and 100 °C in ^1^H NMR. Although a significant decrease occurred, the equilibrium was still favored by the reactants. That result leads to the conclusion that temperatures higher than 100 °C might be necessary for observing considerable retro-Diels–Alder in a material that would react in a suspension in DMSO. The effect of the *endo*/*exo* ratio for that system was discussed, concluding that the *exo* ratio might increase between each cycle that the material undergoes. Furthermore, it was also possible to observe the effect of a longer aliphatic chain on the reactivity of hydroxymaleimides, as well as to satisfactorily determine the kinetic and thermodynamic parameters of the selected molecule pairs.

## Figures and Tables

**Table 1:** Diels–Alder kinetic and thermodynamic constants for reaction FAL–NHM.

Technique	Temp. (°C)	*k_f_* × 10^−3^ (L/mol.s)	*k_r_* × 10^−5^ (s^−1^)	*K_eq_* (L/mol)	SSR
**UV-Vis**	24	0.46	0.02	2478	2.0 × 10^−7^
	37	0.37	0.03	1127	2.5 × 10^−6^
	57	2.24	0.60	374	2.0 × 10^−8^
	76	2.38	1.05	226	7.0 × 10^−6^
	93	16.06	23.23	69	2.1 × 10^−6^
	119	18.21	71.79	25	8.0 × 10^−8^
**NMR**	23	0.20	0.01	2655	4.8 × 10^−4^
	40	0.46	0.03	1832	5.8 × 10^−4^
	60	1.40	0.32	442	2.0 × 10^−5^
	80	4.58	3.19	144	2.3 × 10^−4^
	100	9.87	9.18	107	5.5 × 10^−4^

**Table 2:** Diels–Alder kinetic and thermodynamic constants for reaction of furfuryl alcohol/*N*-(2-Hydroxyethyl)maleimide (FAL–N2HM).

Technique	Temp. (°C)	*k_f_* × 10^−3^ (L/mol.s)	*k_r_* × 10^−5^ (s^−1^)	*K_eq_* (L/mol)	SSR
UV-Vis	57	0.79	0.30	262	3.2 × 10^−6^
	76	1.39	0.97	144	6.7 × 10^−6^
	93	4.10	1.90	211	7.6 × 10^−6^
	119	19.91	19.0	104	7.0 × 10^−7^
NMR	60	0.50	2.02	25	5.6 × 10^−4^
	80	0.90	7.19	12	1.3 × 10^−3^
	100	1.72	19.42	9	2.6 × 10^−4^

**Table 3:** Activation energy, enthalpy, and entropy of formation by reaction and technique.

Reaction	Technique	*E_a_* (kJ/mol) Forward Reaction	*E_a_* (kJ/mol) Reverse Reaction	Δ*H* (kJ/mol)	Δ*S* (J/mol·K)
FAL–NHM	^1^H NMR	48 ± 4	90 ± 15	−43 ± 11	−77 ± 34
	UV-Vis	39 ± 27	88 ± 19	−49 ± 6	−98 ± 17
	Combined	43 ± 7	90 ± 10	−47 ± 5	−91 ± 16
FAL–N2HM	^1^H NMR	32 ± 14	59 ± 14	−27 ± 28	−54 ± 78

## Supplementary Materials

The following are available online. Figure S1: FAL–N2HM conversion versus time (UV-Vis). Figure S2: FAL–NHM conversion versus time. Figure S3: FAL–NHM data fitting from UV-Vis. Figure S4: FAL–NHM data fitting from NMR. Figure S5: Arrhenius plot for FAL–N2HM reaction. Figure S6: Van’t Hoff plot for reaction FAL–N2HM.
