# Supplementary material for: Thermodynamic and Kinetic Study of Diels–Alder Reaction between Furfuryl Alcohol and N-Hydroxymaleimides—An Assessment for Materials Application
Source: Molecules. 2020 Jan 7;25(2):243. doi: 10.3390/molecules25020243 (PMC7024143; doi:10.3390/molecules25020243)
Supplement: Supplementary file 1 [file molecules-25-00243-s001.pdf]

Supplementary Information

Chemical shifts of reactants and products

Furfuryl alcohol

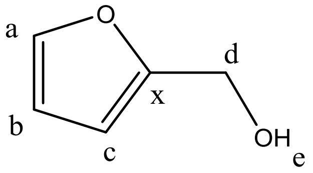

^{1}H NMR (400 MHz, DMSO) δ: 7.56 (dd, 1H, H_a, J = 1.8), 6.38 (dd, 1H, H_b, J = 3.2), 6.27 (dd, 1H, H_c, J = 3.1), 5.17 (t, 1H, H_e, J = 5.8), 4.38 (d, 2H, H_d, J = 5.7).

^{13}C NMR (400 MHz, DMSO) δ: 155.9 (C_x), 142.5 (C_a), 110.7 (C_b), 107.3 (C_c), 56.1 (C_d).

N-Hydroxymaleimide

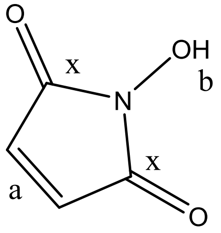

^{1}H NMR (400 MHz, DMSO) δ: 10.34 (s, 1H, H_b), 6.92 (s, 2H, H_a).

^{13}C NMR (400 MHz, DMSO) δ: 167.5 (C_x), 132.6 (C_a).

Adduct 1

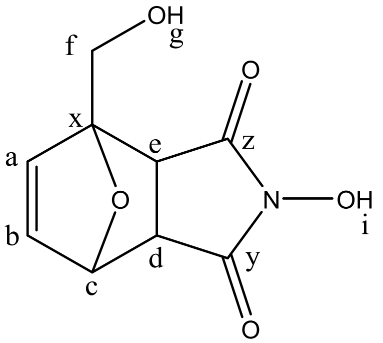

^{1}H NMR (400 MHz, DMSO, endo) δ: 10.66 (s, 1H, H_i), 6.41 (dd, 1H, H_b, J = 5.8), 6.29 (d, 1H, H_a, J = 5.8), 5.25 (dd, 1H, H_c, J = 5.4), 3.96 (dd, 2H, H_f, J = 29.9), 3.55 (dd, 1H, H_d, J = 7.5), 3.32 (d, 1H, H_e, J = 7.5).

^{13}C NMR (400 MHz, DMSO, endo) δ: 171.5 (C_y), 171.3 (C_z), 135.8 (C_b), 135.5 (C_a), 92.6 (C_x), 78.8 (C_c), 60.0 (C_f), 44.6 (C_d), 42.1 (C_e).

^{1}H NMR (400 MHz, DMSO, exo) δ: 10.82 (s, 1H, H_i), 6.54-6.49 (m, 2H, H_a and H_b), 5.07 (d, 1H, H_c, J = 1.4), 3.86 (dd, 2H, H_f, J = 12.41), 2.94 (d, 1H, H_d, J = 6.5), 2.81 (d, 1H, H_e, J = 6.4).

^{13}C NMR (400 MHz, DMSO, exo) δ: 172.5 (C_y), 171.3 (C_z), 138.1 (C_b), 136.5 (C_a), 91.7 (C_x), 80.1 (C_c), 59.3 (C_f), 46.9 (C_d), 44.9 (C_e).

N-(2-Hydroxyethyl)maleimide

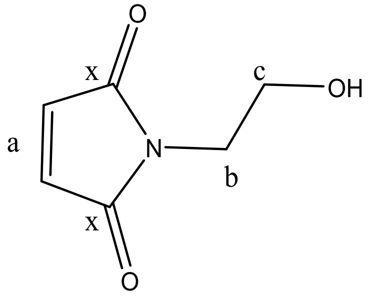

^{1}H NMR (400 MHz, DMSO) δ: 7.00 (s, 2H, H_a), 4.74-4.81 (m, 1H, H_d), 3.44-3.49 (m, 4H, H_b and H_c).

^{13}C NMR (400 MHz, DMSO) δ: 171.6 (C_x), 135.0 (C_a), 58.4 (C_c), 40.4 (C_b).

Adduct 2

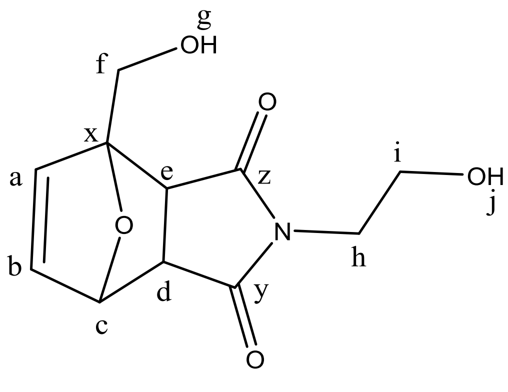

^{1}H NMR (400 MHz, DMSO, endo) δ: 6.39 (dd, 1H, H_b, J = 5.9), 6.28 (d, 1H, H_a, J = 5.8), 5.22 (dd, 1H, H_c, J = 5.5), 3.95 (dq, 2H, H_f, J = 5.6), 3.62 (dd, 1H, H_d, J = 5.6), 3.21-3.44 (m, 5H, H_e, H_h and H_i).

^{13}C NMR (400 MHz, DMSO, endo) δ: 175.8 (C_y), 175.6 (C_z), 135.8 (C_b), 135.4 (C_a), 92.8 (C_x), 79.0 (C_c), 60.0 (C_f), 57.6 (C_i), 48.1 (C_d), 45.6 (C_e), 40.6 (C_h).

^{1}H NMR (400 MHz, DMSO, exo) δ: 6.55-6.49 (m, 2H, H_a and H_b), 5.07 (d, 1H, H_c, J = 1.3), 4.03 (dd, 1H, H_f, J = 6.0), 3.68 (dd, 1H, H_f, J = 7.1), 3.21-3.44 (m, 4H, H_h and H_i) 3.04 (d, 1H, H_d, J = 6.4), 2.87 (d, 1H, H_e, J = 6.4).

^{13}C NMR (400 MHz, DMSO, exo) δ: 176.9 (C_y), 175.4 (C_z), 138.6 (C_b), 136.9 (C_a), 92.1 (C_x), 80.7 (C_c), 59.4 (C_f), 57.8 (C_i), 50.4 (C_d), 48.3 (C_e), 41.0 (C_h).

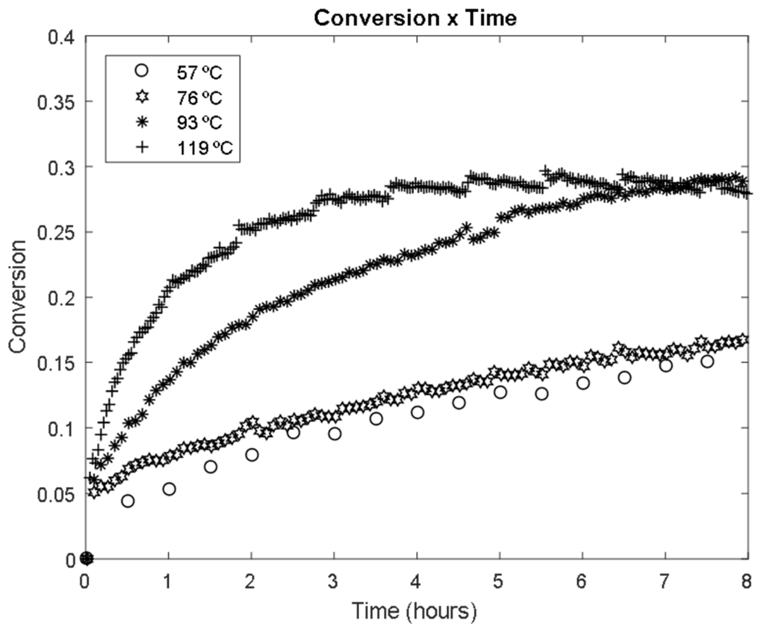

Figure S1. FAL-N2HM conversion *versus* time (UV-Vis)

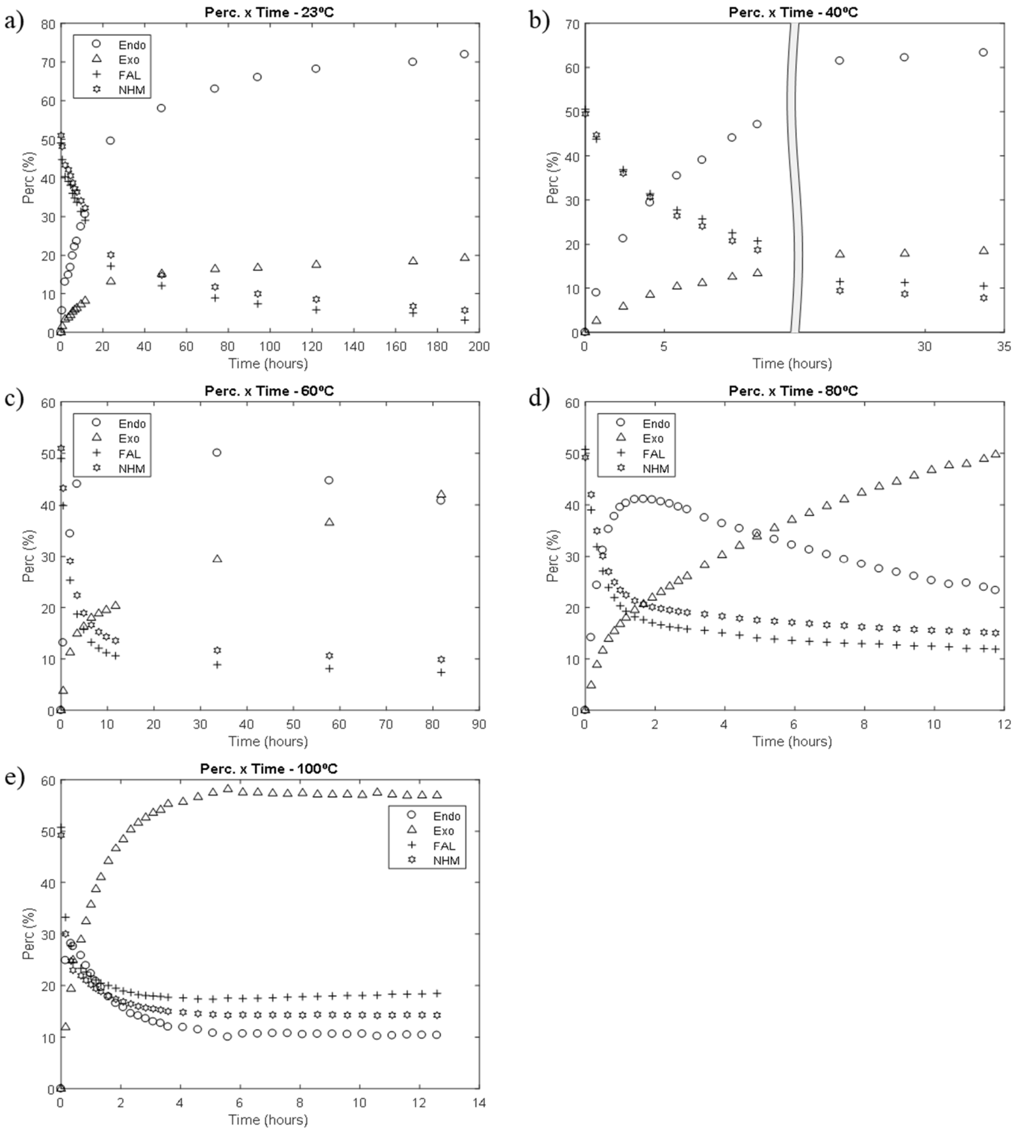

Figure S2. FAL-NHM conversion *versus* time at: a) 23 ºC, b) 40 ºC, c) 60 ºC, d) 80 ºC and e) 100 ºC.

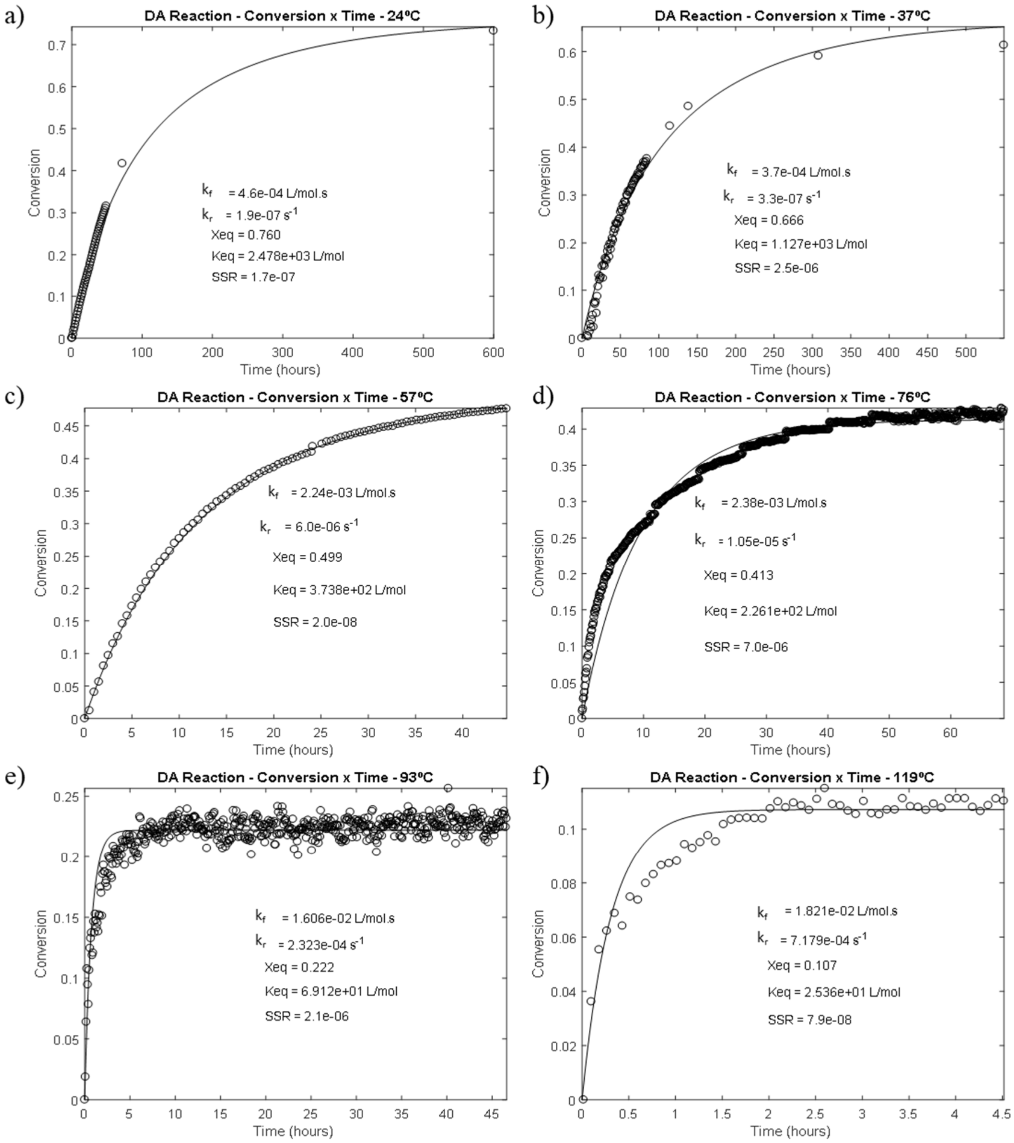

Figure S3. FAL-NHM data fitting from UV-Vis at: a) 24 ºC, b) 37 ºC, c) 57 ºC, d) 76 ºC, e) 93 ºC and f) 119 ºC.

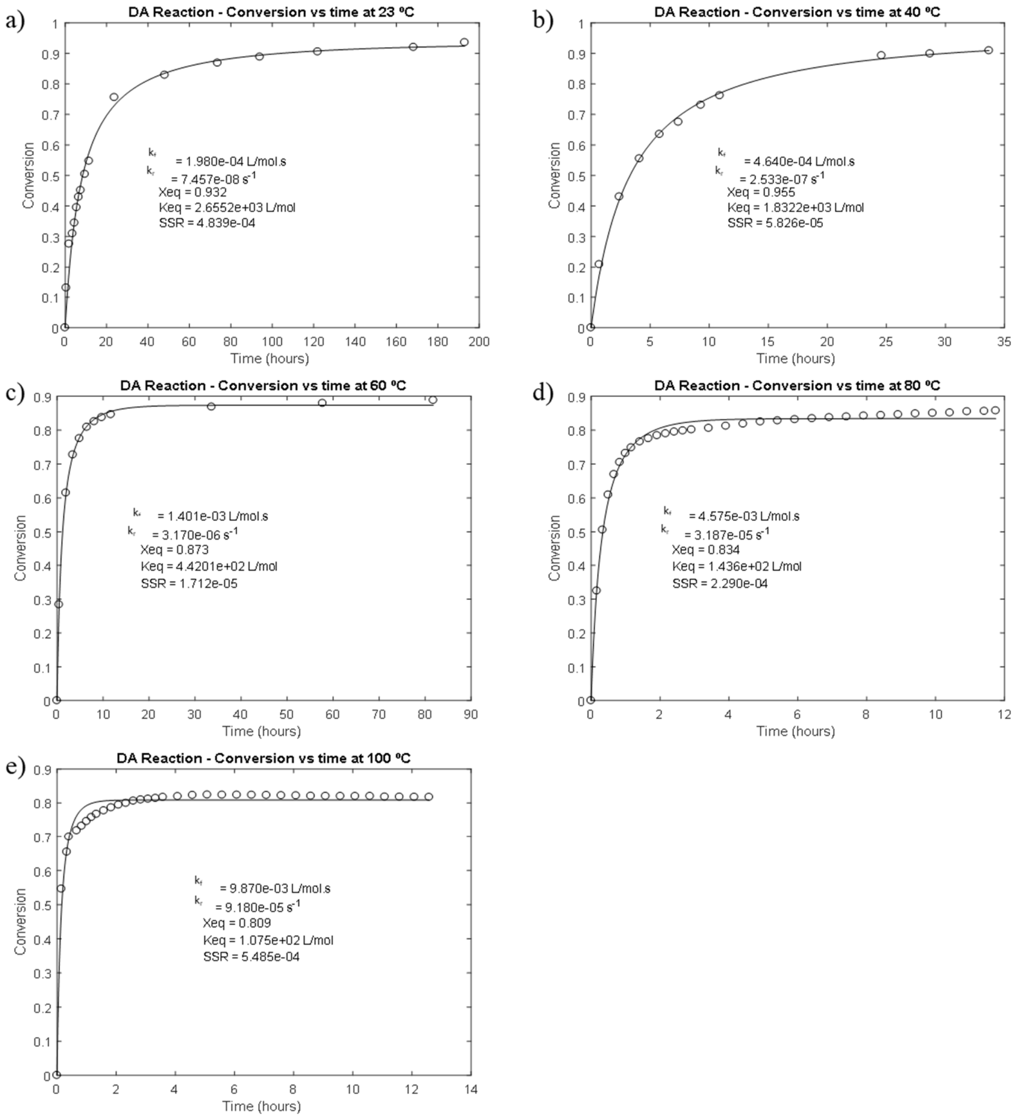

Figure S4. FAL-NHM data fitting from NMR at: a) 23 ºC, b) 40 ºC, c) 60 ºC, d) 80 ºC and e) 100 ºC.

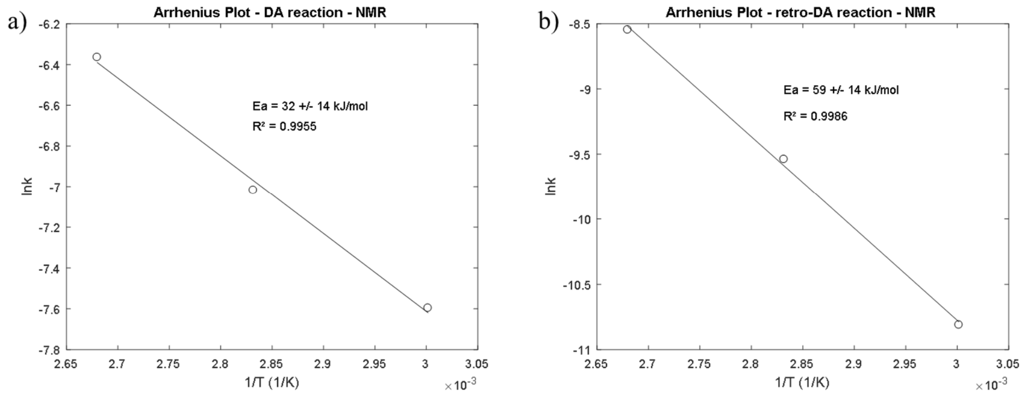

Figure S5. Arrhenius plot for FAL-N2HM reaction: a) direct reaction and b) inverse reaction.

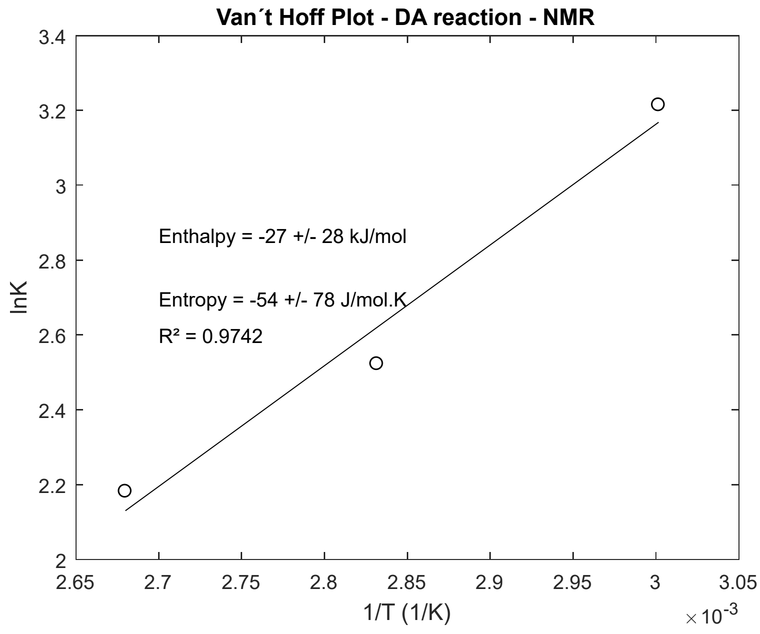

Figure S6. Van't Hoff plot for reaction FAL-N2HM
